# Predictive factors and outcomes of respiratory syncytial virus infection among patients with respiratory failure

**DOI:** 10.3389/fmed.2023.1148531

**Published:** 2023-03-27

**Authors:** Surat Tongyoo, Thummaporn Naorungroj, Jakkrit Laikitmongkhon

**Affiliations:** ^1^Department of Medicine, Faculty of Medicine Siriraj Hospital, Mahidol University, Bangkok, Thailand; ^2^Division of Critical Care, Department of Medicine, Faculty of Medicine Siriraj Hospital, Mahidol University, Bangkok, Thailand

**Keywords:** respiratory syncytial virus, respiratory failure, mechanical ventilation, mortality, critically ill adult patients

## Abstract

**Introduction:**

Respiratory syncytial virus (RSV) infection is an emerging infectious disease. However, the impacts of RSV infection among patients with respiratory failure have not been identified.

**Objective:**

This study investigated the 28-day mortality and clinical outcomes of RSV infection in patients with respiratory failure.

**Methods:**

This retrospective study enrolled patients admitted with respiratory failure and requiring mechanical ventilator support for more than 24 h at Siriraj Hospital, Bangkok, Thailand, between January 2014 and July 2019. Respiratory samples of the patients were examined to identify RSV infections. The primary outcome was 28-day mortality.

**Results:**

Respiratory syncytial virus infection was identified in 67 of the 335 patients with respiratory failure enrolled in this study. There were no significant differences in the following baseline characteristics of the patients with and without RSV infection: mean age (72.7 ± 12.7 years vs. 71 ± 14.8 years), sex (male: 46.3% vs. 47.4%), comorbidities, and initial Murray lung injury scores (1.1 ± 0.8 vs. 1.1 ± 0.9). The 28-day mortality was 38.8% (26/67) for the RSV group and 37.1% (99/268) for the non-RSV group (*p* = 0.79). However, the RSV group had significantly higher proportions of bronchospasm (98.5% vs. 60.8%; *p* < 0.001), ventilator-associated pneumonia (52.2% vs. 33.8%; *p* = 0.005), and lung atelectasis (10.4% vs. 3.0%; *p* = 0.009) than the non-RSV group.

**Conclusion:**

Among the patients with respiratory failure, the 28-day mortality of patients with and without RSV infection did not differ. However, patients with RSV infection had an increased risk of complications, such as bronchospasm, ventilation-associated pneumonia, and lung atelectasis.

## Introduction

Respiratory syncytial virus (RSV) is a common pathogen in children and adults with upper and lower respiratory tract infections. RSV is associated with increased morbidity and mortality (1–4). Moreover, the clinical presentations of RSV vary. They encompass the mild symptoms of upper respiratory tract infections (running nose and symptoms of the common cold or flu) in children and adult or symptoms of severe lower respiratory tract infections (acute bronchitis, pneumonia, and acute respiratory distress syndrome [ARDS]) in premature and elderly age over 65 years old. The development of acute respiratory failure requiring mechanical ventilator support has been variously reported for 10 to 30% of patients with RSV infection, especially high-risk patients (1, 5, 6). The mortality rate of such patients was relatively higher than that of general respiratory patients from other causes (1, 2). However, a study comparing the mortality rates of hospitalized patients with and without RSV infections in different years would be problematic because of the continual advances in critical care medicine (2). Moreover, there is a paucity of studies on the clinical outcomes, complications, and predictive factors for patients infected with RSV who develop respiratory failure, especially adult patients. In addition, the clinical outcomes of such patients might differ from cases involving respiratory failure from other causes.

We investigated the clinical outcomes, treatments, and respiratory complications of patients with respiratory failure and RSV infection. To determine the influence of RSV infection on outcomes, we compared the outcomes of adult patients requiring respiratory support who had and did not have RSV infection during the same period. We hypothesized that clinical outcomes, treatments, and respiratory complications would differentiate the 2 groups of patients. We also assessed clinical predictive factors to predict hospital mortality.

## Materials and methods

### Study design

The single-center, retrospective, matched cohort study was conducted in a tertiary-care, university-affiliated teaching hospital (Siriraj Hospital, Bangkok, Thailand). Before the research began, the Siriraj Institutional Review Board approved its protocol (539/2562 [EC1]).

### Patients

Patients admitted to the medical ward with respiratory failure and requiring mechanical ventilation support for more than 24 h between January 2014 and July 2019 were considered for screening. We enrolled patients aged at least 18 years who had respiratory samples analyzed to identify respiratory pathogens and RSV infections. The patients were classified as having RSV infection if respiratory pathogen testing was positive through either RSV antigen detection or the real-time reverse transcriptase-polymerase chain reaction technique for RSV RNA. Patients without such information were excluded from the final analysis.

### Sample size

We performed a matched case–control analysis (unmatched 1:4; *Z* = 1.96) using a probability (*P*) value of 0.05 and a power (1-β) of 80%. The sample size was calculated with the nQuery program. At least 67 and 268 patients were needed in the RSV and non-RSV groups, respectively, which meant that more than 335 patients were required.

### Data collection

The patients’ electronic medical records were reviewed to collect details of their baseline characteristics, clinical data, disease severity, hospital courses, and outcomes. The baseline characteristics were age, sex, body mass index, and comorbidities. The clinical data were the respiratory failure types and concurrent pulmonary infections. We selected the worst clinical and laboratory parameter values within 72 h after developing respiratory failure to represent the severity of the disease. Disease severity was determined *via* the initial Murray lung injury score, PaO_2_/FiO_2_ ratio [P/F ratio], positive end-expiratory pressure, dynamic lung compliance and chest X-ray imaging, ARDS, and shock. Hospital courses were defined by treatment and pulmonary complications.

### Definition

Acute respiratory failure was defined as the inability of patient’s respiratory system to meet the requirements of the patient, in terms of oxygenation, ventilation, and metabolic demand (7). Ventilator-associated pneumonia (VAP) is defined as pneumonia that occurs 48 h or after, post-endotracheal intubation (8). Healthcare-associated pneumonia (HAP) was defined as pneumonia that occurs 48 h or more after admission and community-acquired pneumonia (CAP) was defined according to the Infectious Diseases Society of America/American Thoracic Society (IDSA/ATS) criteria for community-acquired pneumonia (9, 10).

### Microbiological diagnoses

All samples from nasopharyngeal wash, throat swab, sputum, endotracheal aspirate, or bronchoalveolar lavage of the patients were sent to the diagnostic laboratory, Department of Microbiology, Faculty of Medicine, Siriraj Hospital to detect RSV and bacterial co-infection. Laboratory analysis for detection of RSV was performed using real-time RT-PCR and immunofluorescence assay (IFA) according to the standard protocols. The NucliSENS®easyMag® nucleic acid extraction platform (bioMérieux, Marcy-l’Étoile, France) was used to extract RSV RNA from 200 μl of patient respiratory sample. RSV RNA was eluted with 80 μl elution buffer before being identified with the AllplexTM Respiratory Panel 1A Assay (Seegene, Seoul, South Korea). Amplification was performed on a CFX96 Touch Real-Time PCR Detection System (Bio-Rad Laboratories, Hercules, CA, United States). The result was automatically interpreted by Seegene Viewer software (Seegene). The IFAwas performed using a monoclonal antibody specific to RSV (cat.# 5,006, Millipore, United States) and antimouse immunoglobulin labeled with FITC (cat.# 5,008, Millipore).

In order to identify bacterial co-infection, all sputum samples were evaluated for quality by direct Gram stain. Due to the possibility of oral microbiota contamination, sputum samples with profuse squamous epithelial cells or squamous epithelial cells ≥10/low power field (10X) were not sent for bacterial culture. Endotracheal aspirate samples, which are typically obtained through deep tracheal suction, and bronchoalveolar lavage were accepted for bacterial culture. All samples were inoculated onto sheep blood, MacConkey, and chocolate agars. Agar plates were incubated at 35°C in a 5% CO_2_ atmosphere and examined daily for bacterial isolate growth. Bacterial identification and quantification of the isolates were performed after 18–24 h of incubation. According to the local protocol, a combination of conventional biochemical testing and automated phenotypic identification systems (Vitek-2) (bioMérieux, Durham, NC, United States) was used to identify the potential bacterial pathogen. Antimicrobial susceptibility tests (ASTs) were performed using the disk diffusion method and commercial broth microdilution methods or Vitek-2 (bioMérieux, Durham, NC) based on the type of isolate and the local laboratory protocol. The Clinical and Laboratory Standards Institute (CLSI) interpretative breakpoint criteria were used to interpret and define the phenotypic AST profile of the bacterial isolate.

### Outcome assessments

The primary outcome of the study was 28-day mortality. The secondary outcomes were ventilator-dependent days, hospital length of stay, tracheostomy after respiratory failure, and hospital death. The ventilator-dependent days were calculated from the first day that the patients were intubated until extubation. In cases of death, the ventilator days were the number of days from the first day of mechanical ventilation support until death.

### Statistical analysis

We matched patients in the RSV group with respiratory failure patients confirmed as RSV negative (the control group) by matching their baseline characteristics (age, sex, year of hospitalization, and admission ward). Continuous variables are presented as either the means ± standard deviation (compared using Student’s *t*-test) or as medians ± interquartile range (compared using Wilcoxon rank-sum test). Categorical variables were presented as numbers and percentages, with differences between groups analyzed using Fisher’s exact tests. We performed univariate and multivariate analyses to identify predictive factors associated with the in-hospital death of patients with acute respiratory failure. Their results are presented as relative risks (RRs) and corresponding 95% confidence intervals (CIs). The univariate analysis used a *p*-value < 0.15 to identify the potential predictive factors to be enrolled in the multivariate analysis model. The multivariate model also included RSV infection as a factor of interest. All analyses were conducted using PASW Statistics for Windows, version 18.0 (SPSS Inc., Chicago, IL, United States), and *p*-values less than 0.05 were considered statistically significant.

## Results

### Baseline characteristics and clinical parameters

**Figure 1:**
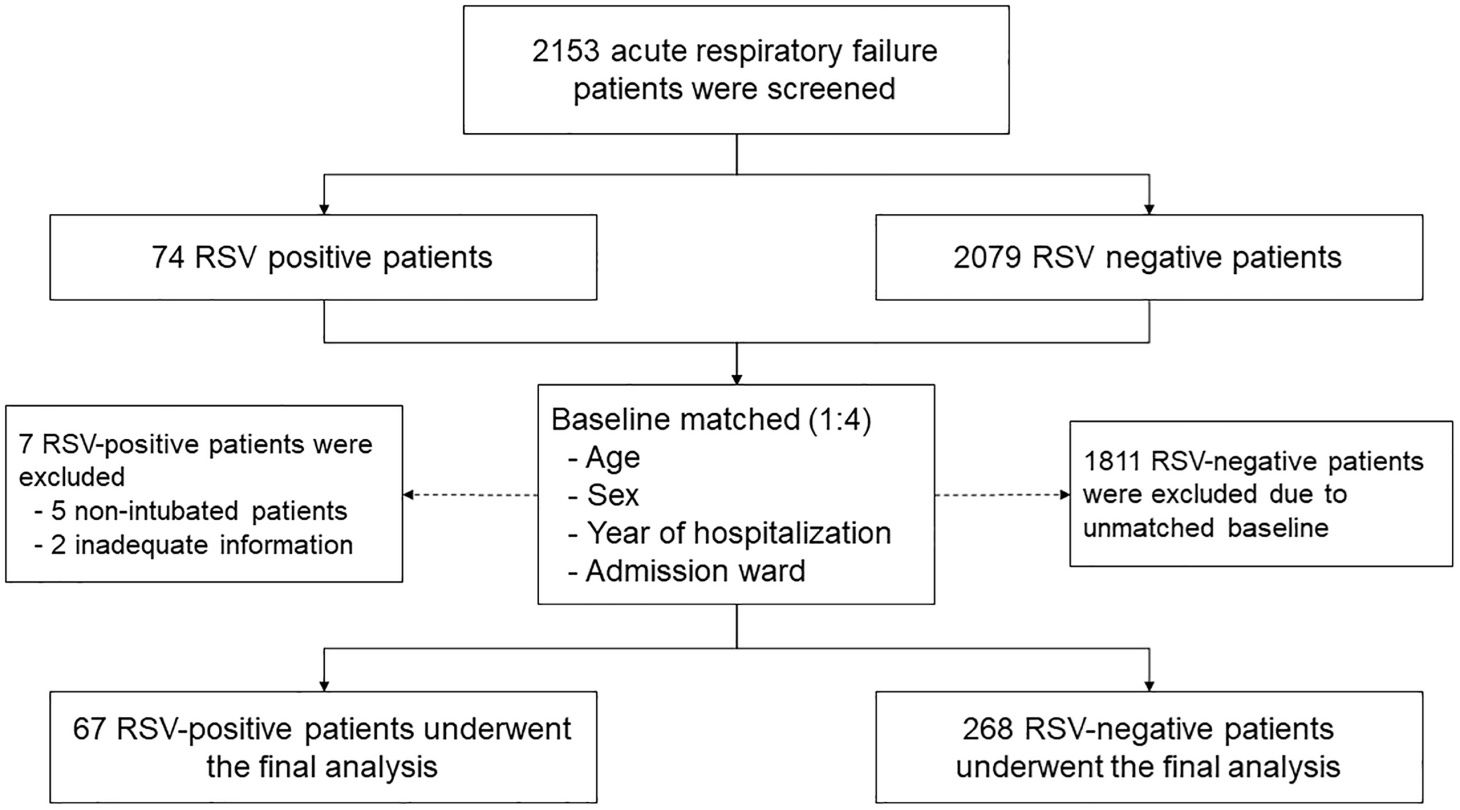
Flow diagram demonstrating the screening and enrollment of patients with and without respiratory syncytial virus infection. RSV, respiratory syncytial virus.

A total of 2,153 patients with respiratory failure were screened, and 74 had confirmed RSV infections. Seven of the 74 patients were excluded because they received noninvasive ventilation or palliative care or their records lacked some information required for the study analysis. Data on 335 patients were used for the final analysis, with 67 patients in the RSV group and 268 in the non-RSV group (Figure 1). There were no significant differences in the baseline characteristics of the RSV and non-RSV groups: mean age, 72.7 ± 12.7 years vs. 71.0 ± 14.8 years; male, 46.3% (31/67) vs. 47.4% (127/268); and BMI, 22.2 ± 5.2 vs. 23.4 ± 6.4, respectively (Table 1). The baseline comorbid diseases, smoking history, and respiratory failure types of the groups did not differ. However, community-acquired pneumonia was significantly higher in the RSV group, while healthcare-associated pneumonia was more significant in the non-RSV group. The most common co-pulmonary infection was *Klebsiella pneumoniae, followed by Acinetobacter baumannii and Pseudomonas aeruginosa*. The concomitant respiratory viral infection was 1 case (1.5%) versus 18 cases (6.7%) of influenza viral infection in the RSV and non-RSV group, respectively. There was no detection of parainfluenza nor adenovirus infection among our study population (Table 1). There were no differences in parameters associated with lung severity (Murray lung injury score, lung compliance, P/F ratio, and positive end-expiratory pressure; Table 2).

**Table 1:** Comparison of the baseline characteristics of acute respiratory failure patients with and without respiratory syncytial virus infection.

Baseline parameters	Without respiratory syncytial virus infection (*n* = 268)	With respiratory syncytial virus infection (*n* = 67)	*P*-value
Age (y)	71.0 ± 14.8	72.7 ± 12.7	0.39
Sex (male, %)	127 (47.4)	31 (46.3)	0.87
Body mass index (kg/m^2^)	23.4 ± 6.4	22.2 ± 5.2	0.23
Underlying conditions, *n* (%)			
Hypertension	204 (76.1)	43 (64.2)	0.07
Coronary artery disease	125 (46.6)	28 (41.8)	0.48
Diabetes mellitus	124 (46.3)	24 (35.8)	0.12
Chronic lung disease	90 (33.6)	23 (34.3)	0.91
Ex-smoker	82 (30.6)	20 (30.3)	1.00
Cerebrovascular disease	54 (20.1)	17 (25.4)	0.35
Chronic steroid use	49 (18.3)	11 (16.4)	0.72
Malignancy of solid organs	30 (11.2)	5 (7.5)	0.37
Hematologic malignancy	18 (6.7)	7 (10.4)	0.30
Asthma	17 (6.3)	7 (10.4)	0.29
Current smoking	11 (4.1)	3 (4.5)	1.00
Cause of respiratory failure, *n* (%)			
Community-acquired pneumonia	227 (84.7)	50 (74.6)	0.05
Healthcare-associated pneumonia	41 (15.3)	17 (25.4)	0.05
Type of respiratory failure, *n* (%)			
Hypoxemic respiratory failure	121 (45.1)	24 (35.8)	0.17
Hypercapnic respiratory failure	127 (47.4)	40 (59.7)	0.07
Increased metabolic demand	20 (7.5)	3 (4.5)	0.39
Co-pulmonary infection, *n* (%)			
Gram positive bacteria			
*Staphylococcus aureus*	13 (4.9)	7 (10.4)	0.14
*Streptococcus pneumoniae*	3 (1.1)	1 (1.5)	1.00
Other Streptococcus species	12 (4.5)	3 (4.5)	1.00
Gram negative bacteria			
*Klebsiella pneumoniae*	29 (10.8)	7 (10.4)	1.00
*Acinetobacter baumannii*	24 (9.0)	9 (13.4)	0.27
*Pseudomonas aeruginosa*	23 (8.6)	9 (13.4)	0.23
*Haemophilus influenzae*	10 (3.7)	2 (3.0)	1.00
*Escherichia coli*	9 (3.4)	0 (0)	0.21
Viral infection			
Influenza virus	18 (6.7)	1 (1.5)	0.10

**Table 2:** Comparison of the clinical parameters of acute respiratory failure patients with and without respiratory syncytial virus infection.

Clinical parameters	Without respiratory syncytial virus infection (*n* = 268)	With respiratory syncytial virus infection (*n* = 67)	*P*-value
Parameters associated with lung severity			
Murray lung injury score	1.1 ± 0.9	1.1 ± 0.8	0.77
Lung compliance	31.8 ± 12.9	28.0 ± 8.5	0.10
PaO_2_/FiO_2_ ratio	264.0 ± 106.5	268.5 ± 103.1	0.77
Positive end-expiratory pressure (cmH_2_O)	5.8 ± 1.8	5.6 ± 1.5	0.47
Treatment received during admission, *n* (%)			
Bronchodilator drug	163 (60.8)	66 (98.5)	<0.001
Inhaled steroid	116 (43.3)	35 (52.2)	0.19
Ribavarin	2 (0.7)	54 (80.6)	< 0.001
Renal replacement therapy	36 (13.4)	3 (4.5)	0.12
Tracheostomy	42 (15.7)	7 (10.4)	0.78
Complications, *n* (%)			
Shock requiring vasopressor	97 (36.2)	23 (34.3)	0.78
Ventilator-associated pneumonia	90 (33.8)	35 (52.2)	0.005
Acute respiratory distress syndrome	31 (11.6)	6 (9.0)	0.54
Pleural effusion	29 (10.9)	10 (14.9)	0.36
Lung atelectasis	8 (3.0)	7 (10.4)	0.009
Outcomes, *n* (%)			
28-day mortality	99 (37.1)	26 (38.8)	0.79
Hospital mortality	95 (35.4)	24 (35.8)	0.95
Ventilator-dependent days	20.5 ± 27.5	18.9 ± 20.0	0.62
Hospital-admission days	30.3 ± 29.0	33.9 ± 38.1	0.40

### Hospital courses and outcomes

The 28-day mortality was 38.8% (26/67) for the RSV group and 37.1% (99/268) for the non-RSV group (*p* = 0.79). The groups had no significant differences in their hospital mortality rates, ventilator-dependent days, or hospital lengths of stay. Nonetheless, significantly higher proportions of patients in the RSV group were administered bronchodilator drugs (98.5% [66/67] vs. 60.8% [163/268]; *p* < 0.001) and ribavirin (80.6% [54/67] vs. 0.7% [2/267]; *p* < 0.001). There were no significant differences in the groups’ other treatment modalities (systemic corticosteroids, renal replacement therapy, and tracheostomy). Patients in the RSV group had significantly more ventilator-associated pneumonia (VAP) and lung atelectasis than those in the non-RSV group (52.2% [35/67] vs. 33.8% [90/268], *p* = 0.005; and 10.4% [7/67] vs. 3% [8/268], *p* = 0.009, respectively). However, the groups had similar rates for other complications (ARDS and pleural effusion; Table 2).

### Comparison within the RSV group

The proportions of non-survivors receiving ribavirin and tracheostomy were higher than the corresponding values for survivors (95.8% [23/24] vs. 72.1% [31/43], with *p* = 0.02; and 20.8% [5/24] vs. 4.7% [2/43], with *p* = 0.04, respectively; Table 3). However, the nonsurvivors and survivors had no significant differences in the causes of respiratory failure or their other treatments (bronchodilator drugs, systemic corticosteroids, and vasopressors).

**Table 3:** Comparison of the characteristics of acute respiratory failure patients with respiratory syncytial virus infection who survived to hospital discharge and those who died in hospital.

Clinical parameters, *n* (%)	Hospital survivors (*n* = 43)	Non-survivors (*n* = 24)	*P*-value
Community-acquired RSV infection	30 (69.8)	12 (50.0)	0.11
Hospital-acquired RSV infection	13 (30.2)	12 (50.0)	0.11
Treatment received			
Bronchodilator drug	42 (97.7)	24 (100)	0.96
Ribavirin	31 (72.1)	23 (95.8)	0.02
Inhaled steroid	20 (46.5)	15 (62.5)	0.21
Vasopressor	13 (30.2)	10 (41.7)	0.35
Tracheostomy	2 (4.7)	5 (20.8)	0.04

RSV, respiratory syncytial virus.

### Predictive factors associated with in-hospital death of acute respiratory failure patients

We analyzed clinical parameters to identify predictive factors associated with hospital mortality. The independent predictive factors associated with in-hospital death were ARDS, VAP, and prolonged ventilator support (>14 days). The multivariate analysis determined that the 3 factors were independently associated with increased hospital mortality. Their RR (95% CI) values were as follows: ARDS, 4.25 (1.58–11.42, *p* = 0.004); VAP, 10.21 (4.83–21.59, *p* < 0.001); and prolonged ventilator support, 2.31 (1.03–5.21, *p* = 0.04). On the other hand, tracheostomy was associated with decreased mortality (RR, 0.33 [0.13–0.82]; *p* = 0.02). However, RSV infection (RR, 0.66 [0.31–1.42]; *p* = 0.29) was not associated with increased hospital death. Detailed results of the univariate and multivariate analyses are presented in Table 4.

**Table 4:** Univariate and multivariate analyses to identify predictive factors associated with in-hospital death of acute respiratory failure patients.

Clinical parameters	Univariate analysis: RR (95% CI)	*P*-value	Multivariate analysis: RR (95% CI)	*P*-value
Body mass index <22 kg/m^2^	1.27 (1.05–1.54)	0.009	1.32 (0.71–2.44)	0.38
Malignancy of solid organ	1.42 (1.00–2.05)	0.03	2.49 (0.98–6.29)	0.05
Chronic renal failure	1.65 (0.97–2.96)	0.03	0.62 (0.15–2.59)	0.51
Murry lung injury score > 1	1.21 (1.02–1.44)	0.03	0.57 (0.27–1.18)	0.13
Hypoxemic respiratory failure	1.14 (0.96–1.35)	0.13	1.26 (0.16–9.79)	0.83
Hypercapnic respiratory failure	0.85 (0.72–1.00)	0.05	0.94 (0.12–7.68)	0.95
Respiratory failure from increased metabolic demand	1.49 (0.91–2.47)	0.05	0.82 (0.14–4.95)	0.83
Acute respiratory distress syndrome	1.88 (1.21–2.94)	<0.001	4.25 (1.58–11.42)	0.004
Shock	1.94 (1.53–2.45)	<0.001	1.63 (0.15–18.19)	0.69
Respiratory syncytial virus infection	1.03 (0.83–1.27)	0.78	0.66 (0.31–1.42)	0.29
Pleural effusion	1.40 (1.00–1.99)	0.03	2.28 (0.95–5.51)	0.07
Ventilator-associated pneumonia	2.50 (1.94–3.29)	<0.001	10.21 (4.83–21.59)	< 0.001
Renal replacement therapy	1.63 (1.18–2.26)	<0.001	2.32 (0.95–5.63)	0.13
Ventilator-dependent days >14	1.81 (1.44–2.28)	<0.001	2.31 (1.03–5.21)	0.04
Tracheostomy	1.33 (1.00–1.79)	0.03	0.33 (0.13–0.82)	0.02

CI, confidence interval; RR, relative risk.

## Discussion

### Key findings

We assessed the clinical outcomes of RSV infection in critically ill patients who developed respiratory failure requiring mechanical ventilator support. We found that 28-day mortality, duration of mechanical ventilation support, and length of stay in the intensive care unit were similar to respiratory failure from other causes. However, the clinical complications of bronchospasm, VAP, and lung atelectasis were more prevalent in patients with RSV than in those without the infection. Our multivariable modeling determined that ARDS, VAP, and prolonged ventilator days were independently associated with subsequent in-hospital death, whereas RSV infection and antiviral therapy were not associated with decreased mortality. Tracheostomy was identified as a factor associated with reduced in-hospital mortality.

### Relationship to previous studies

Respiratory syncytial virus is now recognized as a cause of severe health problems, especially among older adults, patients with known cardiopulmonary disease, and immunocompromised patients (11, 12). The mortality among patients with RSV infections has been reported to be high (approximately 40%) (1, 2), with bronchospasm being a common clinical sign of respiratory tract infection (5, 13), which aligns with our results. Moreover, a study reported a mortality rate for patients with RSV infections that was higher than that found by the present study.

Our study determined that hospital mortality, 28-day mortality, ventilator-dependent days, and hospital-admission days were similar for patients with and without RSV infection. There are several possible explanations for this result. First, patients were assigned to the non-RSV group if testing had previously confirmed that they were negative for RSV infection. Consequently, their respiratory failure was primarily pulmonary in origin, especially respiratory tract infections, resulting in a higher mortality rate than general respiratory failure patients. Second, an earlier study enrolled patients admitted to an intensive care unit after developing respiratory failure during the first 24 h of their hospital admission. The patients’ transfers to the intensive care unit meant they gained quick treatment access. In contrast, our study found that only 38.8% (104/268) of our patients with respiratory failure were able to be admitted to the intensive care unit due to bed shortages. Our lower rate of admission to the intensive care unit for patients with respiratory failure requiring mechanical ventilator support could have resulted in an unnecessarily high mortality rate.

We also found that bronchospasm was a relatively common complication in a majority (approximately 70%) of patients with RSV, which is consistent with cohort studies (5, 13). Severe bronchospasm causes difficult weaning and passive lung atelectasis. Thus, either prolonged ventilation or VAP inevitably occurred, both of which are associated with increased mortality.

Currently, no clinical practice guidelines recommend using antiviral therapy (ribavirin) and corticosteroids for the general treatment of RSV other than immunocompromised patients (patients with malignancy, hematologic stem cell transplantation, or a lung transplant) (14). Lee et al. showed that systemic corticosteroid use was not a survival benefit but was independently associated with longer hospitalization durations and higher rates of culture-proven, hospital-acquired bacterial infection (13). Our study confirmed that among the patients with RSV, treatment with the antiviral medication ribavirin was more common in the non-survivor group than in patients who survived to hospital discharge. However, this antiviral was not statistically associated with decreased mortality.

### Study implications

This study indicated that RSV infection is associated with morbidity and mortality in patients with respiratory failure. To ensure prompt treatment if necessary, the patients should be monitored for the respiratory complications of VAP and lung atelectasis, particularly if prolonged mechanical ventilation support is provided. Moreover, this study implied that early weaning should be considered and that tracheostomy should be performed as soon as the need for prolonged airway or mechanical ventilator support is recognized.

### Study strengths and limitations

This study drew upon a sizable cohort and reported the clinical outcomes, complications, and predictive factors of hospitalized patients diagnosed with RSV infection. We focused on patients who had respiratory failure requiring mechanical ventilation. On the other hand, there are several limitations. First, as these results were from an observational study, biases and residual confounders can arise from the nature of the study. However, as we focused on RSV infection in respiratory failure patients, we could identify predictive factors of mortality in such patients. Second, the mortality rate in the non-RSV group might not reflect the rate for respiratory failure patients in general practice. This is because the study’s inclusion criteria restricted enrollment to patients who had received RSV testing, and most of this group had respiratory tract infection as a comorbidity. Third, although the mortality rate depends on many factors in addition to pulmonary severity, other organ failures should be considered.

## Conclusion

The 28-day mortality among respiratory failure patients infected with RSV does not differ from that of patients without RSV infection. However, patients with RSV infection have significantly higher incidences of complications (bronchospasm, VAP, and lung atelectasis).

## Data availability statement

The raw data supporting the conclusions of this article will be made available by the authors, without unduereservation.

## Ethics statement

The studies involving human participants were reviewed and approved by the Siriraj Institutional Review Board approved its protocol (539/2562 [EC1]). Written informed consent for participation was not required for this study in accordance with the national legislation and the institutional requirements.

## Author contributions

ST took responsibility for the study designed, statistical analysis, and manual searches for additional data collection and interpretation, and drafted and revised the manuscript. TN designed the study, performed data analysis, and drafted the manuscript. JL took part in the study design, data collection, and data interpretation and critically reviewed the manuscript. All authors have read and approved the final manuscript and agreed to be responsible for all aspects of the work.

## Conflict of interest

The authors declare that the research was conducted in the absence of any commercial or financial relationships that could be construed as a potential conflict of interest.

## Publisher’s note

All claims expressed in this article are solely those of the authors and do not necessarily represent those of their affiliated organizations, or those of the publisher, the editors and the reviewers. Any product that may be evaluated in this article, or claim that may be made by its manufacturer, is not guaranteed or endorsed by the publisher.
